# Guillain-Barré Syndrome Induced Dysautonomia Resulting in Cardiac Arrest

**DOI:** 10.7759/cureus.12149

**Published:** 2020-12-18

**Authors:** Emily Fryman, Sameer Saleem, Aniruddha Singh

**Affiliations:** 1 Cardiology, University of Kentucky College of Medicine, Bowling Green, USA; 2 Cardiology, The Medical Center at Bowling Green, Bowling Green, USA

**Keywords:** dysautonomia, bradyarrhythmia, ascending paralysis, guillain barre syndrome (gbs), cardiac arrest

## Abstract

Life-threatening cardiovascular complications can occur as a result of Guillain-Barré Syndrome (GBS) induced autonomic dysfunction necessitating the need for early recognition and potential cardiac pacing. We present the case of a 69-year-old female who was admitted to the hospital for worsening fatigue, bilateral lower extremity weakness and inability to ambulate for two days. Five days later, she experienced large fluctuations in blood pressure, appeared diaphoretic, and had spells of bradycardia. This was soon followed by an episode of unresponsive and cardiac arrest with rhythm strip consistent with asystole. Cardiopulmonary resuscitation (CPR) was initiated with a return of spontaneous circulation (ROSC) after 6 minutes. The patient was intubated and transferred to the intensive care unit (ICU). Reassessment of the patient revealed a new bilateral symmetric upper extremity weakness. Respiratory failure with ascending symmetric paralysis warranted a lumbar puncture which revealed albuminocytologic dissociation-ultimately leading to the diagnosis of GBS.

## Introduction

Guillain-Barré Syndrome (GBS) is an acute inflammatory demyelinating disease that affects the peripheral nervous system. It most commonly presents as sudden onset ascending symmetric paralysis. GBS causes autonomic dysfunction in 70% of patients and may present as fluctuations in blood pressure, diaphoresis, changes in gastrointestinal motility, and cardiac arrhythmias [1,2]. Dysautonomia is typically transient and, as a result, is often of minor clinical importance. However, GBS induced severe autonomic instability can result in life-threatening cardiovascular complications and even sudden death in rare cases [3]. Therefore, it is essential to recognize the symptoms of dysautonomia immediately, monitor its course closely, and be vigilant about the risk of sudden and life-threatening cardiac arrest. We present a case of GBS induced dysautonomia that rapidly progressed from bradycardia to asystole; a feared non-shockable form of cardiac arrest.

## Case presentation

A 69-year-old female with a history of hypertension, diabetes mellitus type-2, dyslipidemia and left-sided hemiparesis secondary to a prior ischemic stroke presented with fatigue, progressive bilateral lower extremity weakness and inability to ambulate with walker for two days. She denied any associated symptoms including headache, fever, vision changes, chest pain, dyspnea, abdominal pain, urinary problems or bowel changes.

Initial vital signs included a temperature of 98°F, a blood pressure (BP) of 140/80 mmHg, a heart rate of 80/minute, and oxygen saturation of 99% on room air. Neurologic exam findings included normal speech, no deficits on cranial nerve exam, the patient was alert and orientated to person, place, and date, 3/5 strength in bilateral lower extremities, 5/5 strength in the right upper extremity, and 4/5 strength in left upper extremity.

Initial workup, which included complete blood count (CBC), complete metabolic panel (CMP), vitamin B12, and thyroid-stimulating hormone (TSH) was found to be unremarkable. Serum potassium was normal at 3.5mg/dl. The magnesium level was low at 1.4mg/dl and was replenished. Troponin was negative, and EKG revealed normal sinus rhythm with occasional premature ventricular contractions (PVCs). Computed tomography (CT) scan of the head was negative for an acute ischemic process, haemorrhage or intracranial mass. Spinal stenosis was suspected, and neurosurgery was consulted. Magnetic resonance imaging (MRI) of the thoraco-lumbar spine revealed mild chronic lumbar spinal stenosis, which neurosurgery reported as an unlikely cause of her sudden bilateral leg weakness.

**Figure 1:**
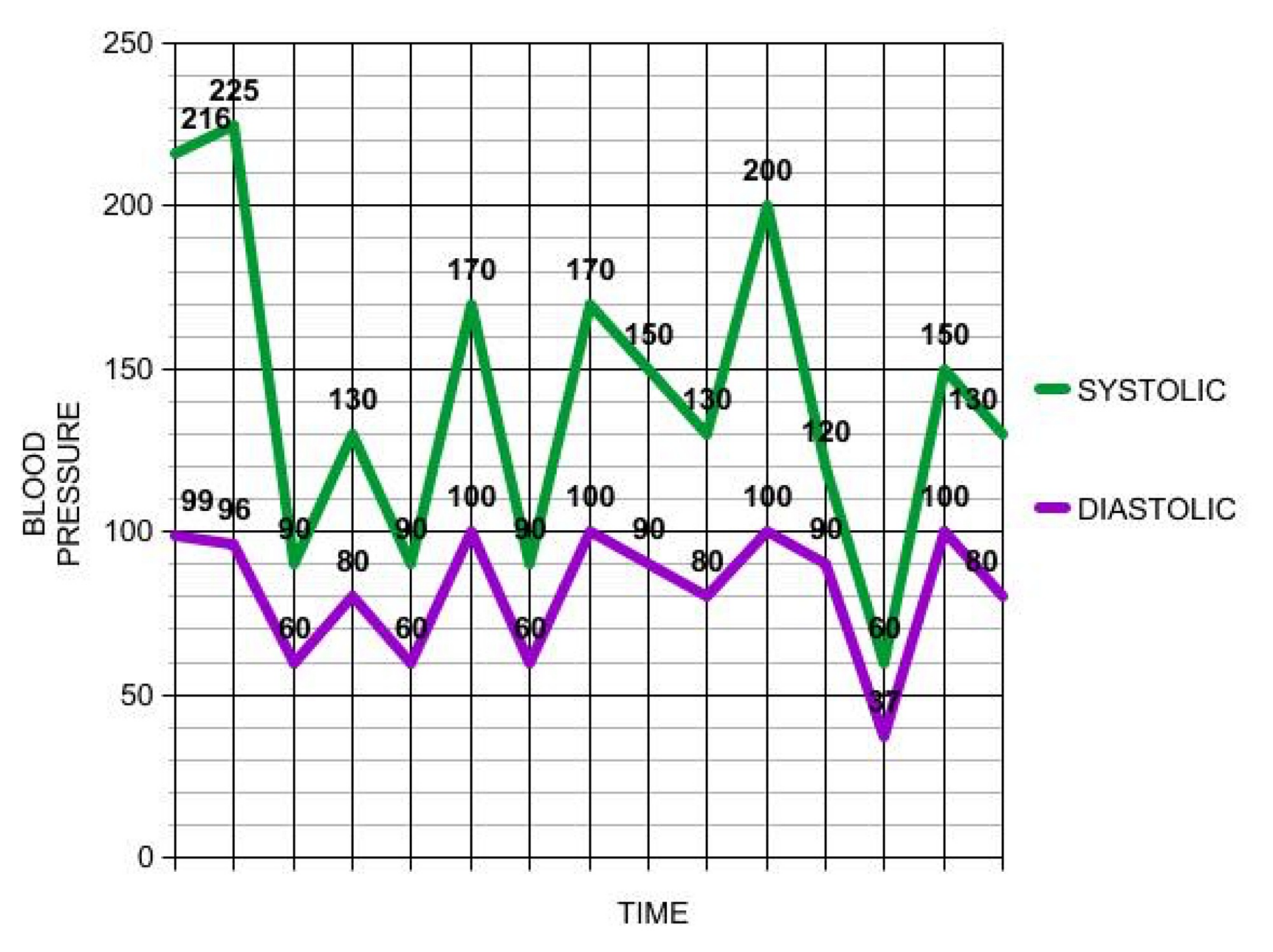
Blood pressure level trend

**Figure 2:**
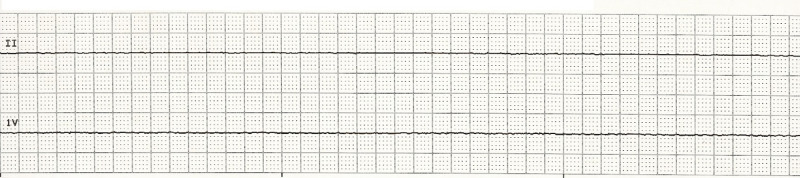
Rhythm strip showing asystole

On hospital day 5, the patient was noted to have profound diaphoresis with fluctuating blood pressure (trend shown in Figure 1), and spells of bradycardia up to 30-40/min. These findings were soon followed by an episode of unresponsiveness with no palpable pulse and telemetry revealing asystole (Figure 2). CPR was initiated, and the patient was given two rounds of epinephrine with ROSC achieved after 6 minutes. The patient was intubated and transferred to the ICU. She was awake following CPR; therefore, hypothermia protocol was not initiated. Stat electrocardiogram (EKG), echocardiogram, CT of the head, and CT angiography of the chest were unremarkable. Blood work was normal, including arterial blood gas, CBC, and CMP. Troponin was mildly elevated at 0.7ng/ml. However, the patient denied any chest pain.

On hospital day six, the patient was unable to pass a continuous positive airway pressure (CPAP) trial. Therefore, mechanical ventilation was continued. Physical exam without sedation revealed paralysis of lower extremities with 0/5 power bilaterally. Patellar reflexes were absent in both knees. Of note, the weakness had now progressed to involve the upper extremities with 2/5 power bilaterally. Given the progression of bilateral ascending weakness and acute respiratory failure, a stat lumbar puncture was performed. Cerebrospinal fluid (CSF) analysis showed glucose of 124mg/dL, a protein of 310mg/dL and a WBC count of 9/mm3, consistent with albuminocytologic dissociation. GBS was suspected, and the patient was started on 400mg/kg/day of intravenous immunoglobulins (IVIGs) for five days. Electromyography and nerve conduction studies (EMG/NCS) were obtained for confirmation of diagnosis, prognosis, and classification, which revealed severe demyelinating polyneuropathy with secondary axonal loss.

After further discussion with the patient’s family members, they reported that the patient had diarrhoea a week before admission that resolved spontaneously. Stool studies were sent for bacteria, viruses, toxins, ova and parasites but were negative. The patient completed five days of IVIG therapy with some improvement. Neurologic exam findings post-IVIG treatment showed 2/5 strength in the feet with 0/5 strength in the legs bilaterally. The right arm and hand had 4/5 strength with the left arm having 1/5 strength. Her left hand had 3/5 strength, which was her baseline strength secondary to residual left-sided hemiparesis. The patient failed several CPAP trials resulting in the continuation of mechanical ventilation. Neurology recommended a tracheostomy and percutaneous endoscopic gastrostomy (PEG) tube insertion, as recovery would likely take months.

On hospital day 12, no further neurologic improvement was documented, and no episodes of bradycardia were noted in the ICU. The patient underwent a tracheostomy and PEG tube insertion without any further complications. Cardiology did not recommend any further cardiac workup for the cardiac arrest. Therefore, the patient was transferred to a long-term acute care facility for further physical rehabilitation.

## Discussion

GBS affects both young people and the elderly with an annual incidence of 1.7 per 100,000 people [4]. While the exact pathophysiology is unknown, the majority recognizes that the immune system is highly involved. A popular theory is that antigens and antibodies cross-react with peripheral nerve epitopes resulting in autoimmunity [4]. There have been reports of several antigenic triggers such as viral infections, bacterial infections, and vaccines. The most commonly reported antecedent infection is with Campylobacter jejuni [5]. Further support of an immune system malfunction comes from the presence of serum anti-GQ1b ganglioside antibodies which has been found in several GBS-variants [5].

The diagnosis of GBS starts with clinical suspicion, followed by CSF analysis. CSF analysis revealing albuminocytologic dissociation (a high protein level with normal WBC count), which is considered pathognomonic for GBS, can be found in up to 66% of cases who present in the first week after the onset of symptoms [5]. Therefore, a normal CSF exam cannot exclude the diagnosis. NCS/EMG can be used to confirm, classify, and determine the prognosis, which will show the presence and degree of nerve conduction deficit. Studies show that 25% of patients with GBS require intubation and mechanical ventilation, with 3% having fatal complications [6].

There is no definitive curative therapy for GBS. However, disease-modifying therapies are effective at decreasing symptom severity and shortening recovery time [5]. The two disease-modifying therapies are plasmapheresis and IVIG. Both options are equally effective, and there is no advantage to combining or sequentially administering these therapies [7]. Plasmapheresis includes 4-6 sessions over an 8-10-day course [7]. The main complications of plasma exchange include hypotension and sepsis [7]. ACE inhibitors should be held 24 hours before the initiating therapy if patients are taking those as there is a risk of anaphylaxis with concurrent ACE inhibitor administration and plasmapheresis [8]. IVIGs are given at 400mg/kg/day for five days [7]. The main complications of IVIG include aseptic meningitis, rash, acute kidney injury, hyperviscosity (rare), and anaphylaxis in the presence of IgA deficiency [7]. For our patient, IgA levels were normal, and IVIG therapy was initiated. Physical therapy is an important factor in the recovery period and should be started as soon as possible.

GBS induced dysautonomia includes blood pressure fluctuations, diaphoresis, GI motility dysregulation, and cardiac arrhythmias [2]. The most common rhythm abnormality associated with GBS dysautonomia is sinus tachycardia [3]. Life-threatening bradyarrhythmias ranging from bradycardia to asystole occur in 7-34% of GBS patients necessitating the need for early recognition and potential cardiac pacing [3]. Bradycardia is likely the result of parasympathetic overstimulation, while sinus arrest might be the result of afferent baroreflex failure [3]. GBS induced bradycardia can occur spontaneously without any warning making it difficult for clinicians to recognize it early hence the need for ICU monitoring. Flachenecker et al. proposed the use of a 24-hour heart rate power spectrum for predicting serious bradyarrhythmias in patients with GBS with a sensitivity of 73% and specificity of 73% [9]. While current literature recognizes bradyarrhythmias as a severe complication of GBS, a definitive management guideline appears to be lacking. Proposed interventions include IV atropine, a temporary pacemaker or a permanent pacemaker [7].

## Conclusions

GBS should be considered in any patient presenting with symmetrical ascending muscle weakness. The diagnosis of GBS requires clinical suspicion followed by CSF analysis, EMG and NCS. A common complication of GBS is autonomic dysfunction, which can range from mild symptoms to life-threatening cardiovascular complications. As discussed above, we present a case of GBS induced bradycardia rapidly progressing to asystole. Therefore, it is important to recognize the symptoms of dysautonomia immediately, monitor its course closely, and be vigilant about the risk of sudden and life-threatening cardiac arrest.
